# Validity of a Self-administered Food Frequency Questionnaire in the 5-year Follow-up Survey of the JPHC Study Cohort I to Assess Sodium and Potassium Intake: Comparison with Dietary Records and 24-hour Urinary Excretion Level

**DOI:** 10.2188/jea.13.1sup_102

**Published:** 2007-11-30

**Authors:** Satoshi Sasaki, Junko Ishihara, Shoichiro Tsugane

**Affiliations:** 1 Epidemiology and Biostatistics Division, National Cancer Center Research Institute East.; 2 Cancer Information and Epidemiology Division, National Cancer Center Research Institute.

**Keywords:** validity, questionnaire, diets, sodium, potassium, urinary excretion

## Abstract

We compared the intake levels of sodium and potassium assessed with a self-administered semiquantitative food frequency questionnaire (FFQ) used in a 5-year follow-up survey of the JPHC study and 28-day dietary record (DR), and the corresponding two 24-hour urinary excretion levels (32 men and 57 women) in 3-areas, i.e., Ninohe, Yokote, and Saku Public Health Center areas. The Spearman rank correlation coefficients between dietary sodium assessed with FFQ and the urinary excretion for crude values were 0.24 and -0.10 in men and women, respectively. After adjusting for energy and creatinine, the sodium correlation coefficients were 0.35 and 0.25 in men and women, respectively. The correlation coefficients for crude potassium values were 0.18 and -0.13 in men and women, respectively. After adjusting for energy and creatinine, the potassium correlation coefficients were 0.48 and 0.18 in men and women, respectively In conclusion, a weak correlation was observed both for sodium and potassium after energy and creatinine adjustment in men, whereas no meaningful correlation was observed in women.

Validation of a newly developed dietary assessment method is important for interpretation of the results of research in which the assessment method is used. Validation using biomarkers is recommended because it can evaluate the validity for target nutrients more objectively than studies with other dietary assessment methods such as the dietary record as the gold standard. Although reliable biomarkers do not exist for many nutrients, some previous studies reported the usefulness of urinary excretions of sodium and potassium as a biomarker for the corresponding intake levels^1^ with some caution.^2^ Two recent validation studies for a newly developed food frequency questionnaire have used sodium and/or potassium as biomarker and found reasonable validity both in Western and Japanese populations.^3^^,^^4^

Sodium and potassium are important nutrients for chronic disease epidemiology on diets because several studies suggested a possible association of the nutrients with mortality from stroke^5^ and cancer of stomach.^6^ Moreover, the international comparative study reported that the 24-hour urinary excretion levels in a Japanese population have been much higher for sodium and much lower for potassium compared to the standards in other developed countries.^7^ Therefore, validation is important for these two nutrients in this newly-developed, self-administered semiquantitative food frequency questionnaire (FFQ) used in a 5-year follow-up survey in the JPHC study. We compared the intake levels of sodium and potassium assessed with the FFQ and 28-day dietary record (DR), and the corresponding 24-hour urinary excretion levels.

## MATERIALS AND METHODS

The study design has been described elsewhere.^8^ Urinary collection was performed in May and November in 3 areas, i.e., Ninohe, Yokote, and Saku Public Health Center (PHC) areas. An analysis was conducted on 89 subjects (32 men and 57 women) with 28-day DR, the FFQ, and two urinary collections from both May and November. For the intake assessed by DR, the mean of 28 days was used. For the urinary excretions, the mean of 2 days was employed. The urine samples were analyzed for Na and K by flame photometry and creatinine by Jaffe's procedure.

Spearman rank correlation coefficients between intakes and urinary excretion were computed, i.e., sodium/potassium intakes assessed by FFQ versus the 24-h urinary excretions, and sodium/potassium intakes assessed by DR versus the 24-h urinary excretions. Intake values were adjusted for energy using a residual model^9^. The urinary excretion values were adjusted for creatinine.^4^

We also performed the same analyses when selecting the subjects with complete urinary collections. The completeness of urinary collection was determined by the following criteria: the creatinine (mg)/body weight (kg) ratio <14.4 or >33.6 in men and <10.8 or >25.2 in women.^10^ The urine collection of 13 men and 24 women (41 and 43%, respectively) was incomplete by the criteria. Since the results were no different whether including and excluding the subjects with incomplete urine collections, the results with all the subjects included were presented.

## RESULTS

Table 1 shows the 24-hour urinary excretion of sodium and potassium by area. Both in men and women, the mean sodium excretion was highest in the Yokote PHC area although the difference was not significant. No apparent area difference was observed for potassium.

**Table 1.** Means±standard deviations of urinary sodium and potassium excretion (two 24-hour urinary collections) (mmol/day) by area

Area	Ninohe PHC area			Yokote PHC area			Saku PHC area			Total			ANOVA
Men	(n=5)			(n=21)			(n=6)			(n=32)			
Sodium	161	±	62	219	±	94	184	±	64	203	±	86	0.755
Potassium	42	±	14	49	±	24	51	±	15	48	±	21	0.507
Women	(n=16)			(n=23)			(n=18)			(n=57)			
Sodium	160	±	67	230	±	67	196	±	76	200	±	74	0.193
Potassium	46	±	11	54	±	19	53	±	19	51	±	17	0.296

Table 2 shows the Spearman rank correlation coefficients between dietary intakes and 24-hour urinary excretions of sodium and potassium. Intakes assessed by two methods, i.e., FFQ and DR, were compared to the urinary excretions of sodium and potassium. Where intakes assessed with the FFQ were concerned, no significant correlation was observed either for sodium or potassium in either crude or energy-adjusted values. When energy- and creatinine-adjusted values were used, a significantly positive correlation was observed for sodium and potassium in men (r=0.35, p<0.05 for sodium and 0.48, p<0.01 for potassium), but not for women. In contrast, the intakes assessed with DR showed a consistently positive correlation with the urinary excretions both in sodium and potassium (r=0.28-0.48 for sodium and r=0.19-0.46 for potassium).

**Table 2.** Sodium and potassium intakes assessed with FFQ and DR (28-day means), their urinary excretion (two 24-hour urine collections), and correlation coefficients

						Spearman correlation with urinary excretion		
	
	Mean	±	Standard deviation	Median	Range	Crude	Energy-adjusted	Energy- and creatinine-adjusted
Men (n=32)								
Dietary Na by FFQ	262	±	123	230	122 - 697	0.24	0.06	0.35^1^
Dietary Na by DR	241	±	44	237	134 - 339	0.41^1^	0.28	0.38^1^
Urinary Na	203	±	86	210	53 - 418	---	---	---
Urinary Na/dietary Na (FFQ)	0.89	±	0.44	0.84	0.11 - 1.90	---	---	---
Urinary Na/dietary Na (DR)	0.85	±	0.36	0.82	0.21 - 1.92	---	---	---
Women (n=57)								
Dietary Na by FFQ	262	±	118	231	55 - 753	-0.10	0.02	0.25^4^
Dietary Na by DR	217	±	39	216	142 - 301	0.41^2^	0.48^3^	0.47^3,4^
Urinary Na	200	±	74	194	55 - 405	---	---	---
Urinary Na/dietary Na (FFQ)	0.92	±	0.56	0.80	0.20 - 3.20	---	---	---
Urinary Na/dietary Na (DR)	0.92	±	0.32	0.88	0.27 - 1.86	---	---	---
Men (n=32)								
Dietary K by FFQ	83	±	33	79	36 - 196	0.18	0.17	0.48^2^
Dietary K by DR	83	±	16	82	56 - 125	0.46^2^	0.29	0.46^2^
Urinary K	48	±	21	47	10 - 110	---	---	---
Urinary K/dietary K (FFQ)	0.65	±	0.32	0.59	0.07 - 1.39	---	---	---
Urinary K/dietary K (DR)	0.58	±	0.25	0.54	0.12 - 1.43	---	---	---
Women (n=57)								
Dietary K by FFQ	89	±	40	79	37 - 244	-0.13	0.00	0.18^4^
Dietary K by DR	79	±	13	78	48 - 113	0.26	0.19	0.30^1,4^
Urinary K	51	±	17	51	16 - 109	---	---	---
Urinary K/dietary K (FFQ)	0.68	±	0.36	0.62	0.19 - 1.62	---	---	---
Urinary K/dietary K (DR)	0.66	±	0.22	0.60	0.21 - 1.37	---	---	---

Significance from null correlation: ^1^ p<0.05, ^2^ p<0.01, ^3^ p<0.001.

^4^n=56.

## DISCUSSION

This study compared dietary intakes and urinary excretions of sodium and potassium. Dietary intake was estimated by 2 types of dietary assessment method: DR and FFQ. Urinary excretions were measured by 24-hour urine samples. The correlation coefficients between dietary intakes and urinary excretion were computed.

The Pearson correlation coefficient between dietary sodium intake assessed by the self-administered diet history questionnaire developed in Japan and the single urinary excretion was 0.09 and 0.16 for crude values and 0.14 and 0.23 for energy- and creatinine-adjusted values in 154 men and 69 women, respectively,^4^ One Finnish study reported a weak correlation between single 24-hour urinary sodium excretion and the salt index calculated from a developed short dietary questionnaire (r=0.18 in 655 men, and r=0.20 in 608 women).^11^ The results of these two studies suggested the difficulty of sodium intake estimation by questionnaire. Even in the studies with dietary record or recall to assess dietary sodium, the correlation between the intake and the urinary excretion varied from 0.04 to 0.91,^3^^,^^12^^-^^16^ indicating that 24-hour urinary excretion is not necessarily a reliable biomarker, probably because of the considerable day-to-day variation in the excretion into urine.^2^ Considering the methodological difficulty to use 24-hour urinary sodium excretion as the gold standard, the results observed in this study (r=0.35 in men and 0.25 in women), when energy- and creatinine-adjusted values were used, indicate a reasonable level of validity for assessment of sodium intake. The correlation coefficient between dietary sodium assessed with 28-day DR and the urinary excretion was 0.41 and 0.41 for crude values and 0.38 and 0.47 for energy- and creatinine-adjusted values in men and women, respectively. These results suggested that the urinary sodium excretions used in this study might be representative at an individual level, although only 2-day urinary collection was used.

We used creatinine as well as energy-adjusted values for correlation analysis (Table 2) in order to minimize the problems due to the uncertainty of 24-hour urinary collection. The correlation improved after adjusting for creatinine.

Spearman rank correlation coefficients between dietary potassium assessed with FFQ developed in the UK and the 2 urinary excretions were 0.25 for crude values and 0.41 for energy-adjusted values in 156 women.^3^ The Pearson correlation coefficient between dietary potassium assessed with a self-administered diet history questionnaire developed in Japan and the single urinary excretion was 0.35 and 0.28 for crude values and 0.34 and 0.40 for energy-adjusted values in 154 men and 69 women, respectively.^4^ Compared to these previous studies, the level of correlation coefficients observed in the present study was somewhat lower except for r=0.48 in men when energy- and creatinine-adjusted values were used.

In conclusion, the level of correlation between the sodium and potassium intakes assessed with FFQ and the corresponding urinary excretions was moderate in men and low in women. More subjects and a longer period of urinary collection may be needed to examine the validity of FFQ when urinary excretion of sodium and potassium is used as the gold standard.
